# Individual factors associated with workaholism among Tunisian engineers

**DOI:** 10.1192/j.eurpsy.2022.350

**Published:** 2022-09-01

**Authors:** A. Bouaziz, R. Sallemi, M. Bouhamed, F. Guermazi, I. Feki, J. Masmoudi

**Affiliations:** 1 Hospital university of HEDI CHAKER, Psychiatry A Department, Sfax, Tunisia; 2 Hedi chaker hospital, Psychiatrie, Sfax, Tunisia

**Keywords:** work addiction, individual factors, Prevalence, engineers

## Abstract

**Introduction:**

Globalization and increased competition in the engineering profession induce to work longer and harder, which predisposes to workaholism or work addiction. Identifying individual factors associated with workaholism could help to maintain the mental health at work.

**Objectives:**

The aim of this study was to assess the prevalence of workaholism and its associated socio-demographic and historic factors among Tunisian engineers.

**Methods:**

A cross-sectional descriptive and analytical study conducted among Tunisian engineers during July 2021. The data were collected by an online questionnaire including the socio-demographic and historic information and the “the Work Addiction Risk Test” (WART) which was used to assess the workaholism.

**Results:**

A total of 52 engineers participated in this study (40.4% female and 59.6% male). The average age was 30.75 years (SD=6.25 years). Concerning marital status, thirty-five engineers (67.3%) were single. Of the participants, 17.3% had a history of chronic somatic-disorders and 25 % of them had a history of a psychiatric disorder, such as depressive disorder in 11.5% of cases. The prevalence of workaholism in Tunisian engineers was 23.1%. Workaholism was associated to older age with no significant difference (p = 0.11). The analysis showed that workaholics had more history of depressive disorder (p = 0.02) compared to non-workaholics. However, no significant difference was found by the other socio-demographic factors according to workaholism.

**Conclusions:**

Workaholism is a significant phenomenon among Tunisian engineers. It may depend of personal characteristics and induce negative consequences on mental health and lead to depression.

**Disclosure:**

No significant relationships.

